# The Role of Language Severity and Education in Explaining Performance on Object and Action Naming in Primary Progressive Aphasia

**DOI:** 10.3389/fnagi.2018.00346

**Published:** 2018-10-30

**Authors:** Marianna Riello, Andreia V. Faria, Bronte Ficek, Kimberly Webster, Chiadi U. Onyike, John Desmond, Constantine Frangakis, Kyrana Tsapkini

**Affiliations:** ^1^Department of Neurology, Johns Hopkins University School of Medicine, Baltimore, MD, United States; ^2^Department of Radiology and Radiological Science, Johns Hopkins University School of Medicine, Baltimore, MD, United States; ^3^Department of Otolaryngology, Head & Neck Surgery, Johns Hopkins University School of Medicine, Baltimore, MD, United States; ^4^Department of Psychiatry and Behavioral Sciences, Johns Hopkins University School of Medicine, Baltimore, MD, United States; ^5^Department of Biostatistics, Johns Hopkins School of Public Health, Baltimore, MD, United States; ^6^Department of Cognitive Science, Johns Hopkins University, Baltimore, MD, United States

**Keywords:** primary progressive aphasia, object naming, action naming, atlas-based analysis, gray matter volumes, education, severity, language severity

## Abstract

Despite the common assumption that atrophy in a certain brain area would compromise the function that it subserves, this is not always the case, especially in complex clinical syndromes such as primary progressive aphasia (PPA). Clinical and demographic information may contribute to PPA phenotypes and explain the manifested impairments better than atrophy. In the present study, we asked how much variance of the object and action naming impairments observed in PPA may be attributed to atrophy in the language network alone vs. additional clinical and demographic factors including language severity and education. Thirty-nine participants with PPA underwent magnetic resonance imaging (MRI) for volumetric analysis and a complete neuropsychological examination, including standardized tests of object and action naming. We used stepwise regression models to compare atrophy (*volumetric* model) to clinical/demographic variables (*clinical-demographic* model) for naming objects and actions. The *clinical-demographic* model was the best-fit model that explained the largest amount of variance in both object and action naming. Brain volume measurements alone explained little variance in both object and action naming. Clinical factors, particularly language severity, and demographic factors, particularly education, need to be considered in conjunction with brain volumes in PPA. The present study emphasizes the complexity of PPA as a syndrome and provides an example of how volumetric, clinical and demographic factors may interact in determining naming performance/deterioration.

## Introduction

It is usually the case that brain volumes correlate with, predict or determine naming performance (Schwartz et al., 2009; Tsapkini et al., 2011); however, this assumption may not hold true for patients with primary progressive aphasia (PPA), given the existing atrophy of many areas in the language network and the relatively high education levels (indicative of cognitive reserve) in these patients. Recently, it has been shown that lower levels of education correlate with greater aphasia severity and performance in a series of tasks ranging from comprehension to production in left-hemisphere stroke patients, even after adjusting for socio-economic status (González-Fernández et al., 2011). Therefore, atrophy may not always correlate with cognitive performance unless other parameters are taken into account, but this has not been explicitly shown in PPA. In the present study, we addressed this issue in the case of object and action naming.

The neural substrates of object and action naming, roughly corresponding to the grammatical categories of nouns and verbs, are extensively studied, especially in post-stroke aphasia (Miceli et al., 1988; Caramazza and Hillis, 1991). Lesion studies have demonstrated that the retrieval of object names is processed in the left anterior and middle temporal cortices, while the equivalent system for verbs is processed in the left frontal regions (Miceli et al., 1988; Damasio and Tranel, 1993; Daniele et al., 1994; Tranel et al., 1997, 2001; Hillis et al., 2002a). More heterogeneous results have been reported in neuroimaging investigations; thus, frontal (Perani et al., 1999; Tranel et al., 2005) and temporal cortices have been involved in verb naming (Perani et al., 1999; Tranel et al., 2005; Benetello et al., 2016), especially in the processing of verb-specific syntactic information (Bedny et al., 2008), and in the lexical processing of active verbs compared to nouns (Yokoyama et al., 2006).

Naming and word-finding difficulties are among the most common deficits in neurodegenerative conditions. Thus, naming tasks are useful tools for the clinician (neurologist, neuropsychologist, or speech-language pathologist). Various types of dementia are increasing in prevalence across aging populations. PPA is an initially focal neurodegenerative syndrome characterized by primary progressive language impairments that eventually affect other cognitive domains and daily functioning (Mesulam, 2007; Gorno-Tempini et al., 2011). According to recent consensus criteria, PPA diagnosis and subtyping include three types of information: imaging data, neuropsychological testing, and clinical features (such as language severity). Within the neuropsychological assessment, naming tests of objects and actions are among the standard neuropsychological assessments used in PPA diagnosis and treatment. Current criteria identify naming problems in spontaneous speech as the core symptoms of the logopenic variant (lvPPA) and semantic variant (svPPA), whereas the non-fluent variant (nfvPPA) may be characterized by impaired speech production or agrammatism (Gorno-Tempini et al., 2011). A pertinent issue in PPA is to determine the brain areas that are responsible for naming performance to inform diagnosis and classification of patients. Studies in PPA indicate that patients with nfvPPA may also show more pronounced oral naming impairment for verbs (Hillis et al., 2002a, 2004; Thompson et al., 2012) than patients with lvPPA (Hillis et al., 2004) and svPPA (Thompson et al., 2012) who may show greater impairments in naming of nouns. A previous study from our group demonstrated a strong correlation between atrophy in the left inferior temporal gyrus (ITG) and naming performance in PPA patients for both objects and actions (Race et al., 2013). The present study seeks to not only explore brain areas that correlate with performance in naming objects and actions but to: (1) quantify the amount of variance explained by the gray matter volumes in naming objects and actions; and (2) evaluate the contribution of clinical and demographic parameters that may explain the variance in object and action naming in PPA.

Several studies have examined the effect of other demographic factors (such as gender or education) on naming performance in neurodegenerative conditions. In patients with neurodegenerative diseases, these studies report an effect of gender (men perform better than women), age, education, IQ, diagnostic groups, hypertension and years post-onset (Randolph et al., 1999; Hall et al., 2012). In PPA only one study looked at the effect of gender, but it showed no difference in performance by gender on the Boston Naming Test (BNT; Rogalski et al., 2007). Given the increasing interest in gender and other demographic and clinical differences in many diseases, especially in brain diseases, as well as the complexity of PPA, it is important to consider their relative contribution in naming performance.

In the present study, we examined the effect of volumetric measures of brain regions in the language network as predictors of object and action naming and compared their predictive value with additional demographic and clinical factors used in PPA clinical assessment. Using stepwise regression models for naming performance on standardized tests of objects and actions, we compared the predictive value of the brain volumes of the language network alone (*volumetric model*) to the additional effects of demographic factors (gender, age, education) and clinical features (years post-onset, severity of language impairment and severity of dementia), hereafter called the *clinical-demographic model*. We also added a secondary analysis introducing the PPA type of variant as a factor, since the three variants have different naming deficits (Gorno-Tempini et al., 2011). In assessing naming performance, we calculated accuracy in lexical access, i.e., word retrieval and phonological representations, rather than motor speech impairments, prevalent in the nfvPPA.

## Materials and Methods

### Participants and Recruitment

Thirty-nine right-handed patients diagnosed with PPA (age range: 50–82 years) participated in the present study. PPA variants were diagnosed according to current diagnostic criteria (Gorno-Tempini et al., 2011). Data were collected from June 2011 to June 2017. All participants had normal or corrected vision; none reported a history of head injury or other neurological problems (other than PPA). Participants were enrolled from Johns Hopkins Outpatient Center’s PPA Clinic or Frontotemporal and Young-Onset Dementia Clinic or referred by physicians specializing in PPA or through clinicaltrials.gov as potential participants for a clinical trial study with a confirmed diagnosis of PPA (ClinicalTrials.gov Identifier: NCT02606422). All participants gave written informed consent and received a thorough language, cognitive and imaging evaluation as part of their participation in the study (see Table 1 for participant characteristics). The experimental procedures and protocol were approved by the Johns Hopkins Hospital Institutional Review Board. All subjects gave written informed consent in accordance with the Declaration of Helsinki.

**Table 1:** Demographic information and cognitive scores (in percentage of correct responses with standard deviations in parentheses) for all the primary progressive aphasia (PPA) patients and variants.

	TOT (*N* = 39)	Lv (*N* = 13)	Nfv (*N* = 18)	Sv (*N* = 8)	*P*
	Mean (SD)	Mean (SD)	Mean (SD)	Mean (SD)	
**Demographic**					
Age	68.28 (7.3)	68.07 (8.9)	68.22 (7.1)	68.75 (5.6)	0.98
Gender	18 F	7 F	6 F	5 F	0.31
Education (yrs)	16.14 (2.6)	15.84 (2.9)	16.88 (2.3)	14.93 (2.1)	0.19
Onset (yrs)	4.13 (2.8)	4.07 (3.1)	3.69 (2.5)	5.21 (3.2)	0.47
Language severity (FTDL–CDR 0–3)	1.79 (0.8)	1.84 (0.8)	1.72 (0.9)	1.87 (0.8)	0.89
Dementia severity (FTDL–CDR 0–24)	6.16 (4.8)	7.57 (4.7)	5.25 (4.6)	5.93 (5.55)	0.41
**Language**					
BNT (30) (%)	51.70 (37.1)	47.69 (33.2)	69.44 (36)	18.33 (26)*	<0.001
HANA (35) (%)	48.93 (35)	42.63 (35.2)	63.65 (34.2)	18.6 (21.3)**	0.002

*Group differences: sv impaired vs. lv; and vs. nfv. **Group differences: sv impaired vs. nfv. Abbreviations: F, female; yrs, years; Lv, Logopenic variant; Nfv, Non-fluent variant; Sv, Semantic variant; BNT, Boston Naming Test; FTLD–CDR, frontotemporal lobar degeneration-Clinical Dementia Rating; HANA, Hopkins Assessment of Naming Actions.

### Clinical Assessment

All patients underwent a complete assessment for demographic and clinical features, and with the Frontotemporal Lobar Degeneration (FTLD)–Clinical Dementia Rating scale (CDR; Knopman et al., 2008) for severity, which provides a semi-quantitative grading of the severity of impairment within a variety of domains. Two severity disease scores were included in the analysis: the total score of the multidimensional evaluation of the FTLD–CDR battery (range 0–24, higher scores indicating more disability)—named “dementia severity” in our study—and the language score at the language subscale of the FTLD–CDR battery (range 0–3)—named “language severity” in our study. Therefore, dementia severity included the sum of the ratings of all the subscales: memory, orientation, judgment and problem-solving, community affairs, home and hobbies, personal care, behavior/comportment, personality and language. Each subscale varied from normal (0) to questionable/very mild (0.5), mild (1.0), moderate (2.0), or severe (3.0) impairment (Knopman et al., 2008). The language subscale particularly differentiates between the following: “normal speech” (0); “minimal but noticeable word-finding problems and non-fluency, with normal comprehension” (0.5); “mild and frequent word-finding problems without degrading spoken speech, or, mild comprehension difficulties” (1); “moderate word-finding problems that interfere significantly with communication, or moderate non-fluency or comprehension in ordinary conversation” (2); “severe deficits in word-finding, in expressive speech and in comprehension making communication nil” (3). This five-level rating scale is efficient in capturing the progression of the overall language impairment that covers all the language deficits characterizing the variants. Furthermore, the language subscale adds unique information in patients with very mild impairment, thus being suitable for the distinction between mild and moderate severity patients (Knopman et al., 2008). Years post-onset were established during the first visit based on the reported history of symptoms.

### Outcome Measures

Object naming was assessed by asking patients to name pictures from the BNT 30-item version (Williams et al., 1989). Action naming was assessed similarly with 35 pictures from the Hopkins Assessment on Naming Actions (HANA; Breining et al., 2015), in which picture names are matching in word frequency to the BNT. Items were considered correct if they were correctly named spontaneously or with a provided semantic cue (but without any phonological cue) according to standard criteria of the BNT manual (Borod et al., 1980; Mack et al., 1992). No cues were given for the HANA, however. Phonological paraphasias were considered errors when the utterances were incomprehensible and unintelligible (usually more than half of the segments of the intended word). Paraphasias due to motor speech deficits were scored as correct as long as they were recognized as the target phonemes, although they could be slightly distorted. In this way we were interested in evaluating factors (i.e., areas of atrophy and demographic and clinical features) that influenced performance on BNT and HANA, taking into account that performance on these tasks represent the result of multiple processes such as: access to meaning, word-retrieval, syntactic and phonological representations excluding motor speech deficits, as previous studies in the field suggest (Mesulam et al., 2013). We focused on *a priori*-identified cerebral regions of interest (ROIs) derived from the relevant literature on PPA atrophy patterns and the language network. Anatomically, lvPPA has been associated with atrophy in the left posterior temporal gyrus, left supramarginal (SMG) and angular gyri (AG; Gorno-Tempini et al., 2004, 2011); nfvPPA has been associated with atrophy in the left inferior frontal gyrus (IFG), left middle frontal gyrus (MFG), dorsal and ventral prefrontal cortex (Gorno-Tempini et al., 2004; Rogalski et al., 2011a); svPPA has been associated with atrophy in the ventral and lateral anterior temporal pole (ATP; Mummery et al., 2000; Hillis et al., 2002b, 2006b), superior temporal gyrus (STG), bilateral anterior ITG (Gorno-Tempini et al., 2004; Rogalski et al., 2011b; Gordon et al., 2016) and bilateral anterior fusiform gyrus (FG).

### MRI Data Acquisition

Most participants underwent magnetic resonance imaging (MRI) the same day of the behavioral evaluation, 11 participants within 2 weeks of the structural brain imaging and three underwent an MRI more than 1 month and fewer than 3 months after the evaluation.

Imaging data were acquired using a 3-T Philips Achieva MRI scanner with a 32-channel head coil. Axial MPRAGE T1–WIs for each participant (TR/TE = 8.1/3.7 ms) were obtained with a 224 × 224 matrix, FOV of 224 × 224 mm and 150 slices of 1.2 mm thickness. The T1-high resolution images were automatically segmented in MRICloud, a public web-based service for multi-contrast imaging segmentation and quantification^1^ (Mori et al., 2016). This process involves orientation and homogeneity correction, two-level brain segmentation (skull-stripping; Tang et al., 2015), then whole brain image mapping based on a sequence of linear, non-linear algorithms, and large deformation diffeomorphic metric mapping (LDDMM; Miller et al., 2005; Wang et al., 2007), and a multi-atlas labeling fusion (MALF; Wang et al., 2013,^2^).

Forty-five atlases (JHU adult atlas, version 9b) were used to generate 289 structural definitions in a five-level ontological hierarchical relationship (Mori et al., 2008, 2013; Oishi et al., 2009). We selected *a priori* ROIs in the language network based on the left-hemisphere atrophy patterns in PPA variants, and included the homologous right-hemisphere areas: bilateral pars opercularis (IFG *opercularis*), pars orbitalis (IFG *orbitalis*), pars triangularis (IFG *triangularis*, Gorno-Tempini et al., 2004; Rogalski et al., 2011b), SMG gyrus (Gorno-Tempini et al., 2004, 2011), temporal pole (TP; Mummery et al., 2000; Hillis et al., 2002b, 2006b), middle temporal gyrus (MTG; Hillis et al., 2002b), ITG (Gorno-Tempini et al., 2004; Rogalski et al., 2011b; Gordon et al., 2016), FG (Gorno-Tempini et al., 2004), dorsolateral prefrontal cortex (DLPFC), ventrolateral prefrontal cortex (VLPFC; Gorno-Tempini et al., 2004; Rogalski et al., 2011b), STG (Gorno-Tempini et al., 2004; Rogalski et al., 2011b; Gordon et al., 2016) and angular gyrus (AG).

All the analyses were performed in participants’ brains’ native space. To control for relative regional atrophy, raw volumes for each ROI were normalized by the total cerebral volume corresponding to the total gray matter volume without myelencephalon and cerebrospinal fluid (CSF). To calculate overall atrophy for each participant while controlling for inter-individual intracranial volume (size of the head), we calculated the ratio between cerebral and intracranial volume (intracranial volume corresponds to the cerebral volume plus the CSF in ventricles and sulci; Zhang et al., 2010). We named this variable *overall atrophy* and added it as a predictor in the regression model.

### Statistical Analyses

Differences between variants on correct production of objects (BNT) and actions (HANA) as within factor were analyzed using repeated measures analyses of variance (ANOVAs). Fisher’s Exact Test for categorical variables (gender) and One-way ANOVA for all the other continuous variables were applied to compare the three PPA variant subgroups’ differences in demographic and clinical features. The alpha level to determine significance was set at *p* < 0.05.

The ability of brain volumes to explain naming behavior was investigated with two separate stepwise multiple regression models. In both models, naming scores for each participant were entered as the dependent variable. The first model (*volumetric*) included the selected 12 left lateralized language areas and their right homologs (24 ROIs) and the overall atrophy as predictors (Tables 2A, 3A). In the second model (*clinical-demographic*), demographic and clinical information were added to the previous model as predictors. The statistical level of significance was calculated in a stepwise fashion for each predictor and set at *p* < 0.05.

**Table 2A:** *Volumetric model on BNT*: naming nouns controlled for normalized volume of the 12 left language areas and their homologs.

Variable	Fraction of std change in BNT, per 1 std change in variable (SE)	*t*-stat	*P*-value	*R*^2^	Added *R*^2^
**Stepwise regression of each ROIs**					
L ITG	0.66 (0.11)	5.92	<0.001	28%	28%
L IFG orbitalis	−0.38 (0.08)	−4.75	<0.001	36%	+8%

*Abbreviations: L ITG, left inferior temporal gyrus; L IFG *orbitalis*, left inferior frontal gyrus pars orbitalis. The Added R-squared from the regression model refers to the additional variance explained by including the given variable*.

**Table 3A:** *Volumetric model on HANA*: naming verbs controlled for normalized volume of the 12 left language areas and their homologs.

Variable	Fraction of std change in BNT, per 1 std change in variable (SE)	*t*-stat	*P*-value	*R*^2^	Added *R*^2^
**Stepwise regression of each ROIs**					
L ITG	0.45 (0.12)	3.69	<0.001	14%	14%
L IFG orbitalis	−0.30 (0.08)	−3.75	<0.001	20%	+6%

*Abbreviations: L ITG, left inferior temporal gyrus; L IFG *orbitalis*, left inferior frontal gyrus pars orbitalis. The Added R-squared from the regression model refers to the additional variance explained by including the given variable*.

## Results

### Descriptive Statistics

The three PPA variant groups did not differ significantly regarding gender, age, education, duration of the disease (years post-symptom onset) and severity ratings (dementia and language severities; see Table 1 for descriptive statistics in each variant).

The ANOVA of the two naming tasks on demographics and variant did not reveal any differences between task (*F*_(2,36)_ = 0.231, *p* = 0.643). Performance in both tasks differed between variants (*F*_(2,36)_ = 5.63, *p* = 0.007); nfvPPA performed better than svPPA (*p* < 0.03 for both tasks).

### Results of Object Naming (BNT)

**Figure 1:**
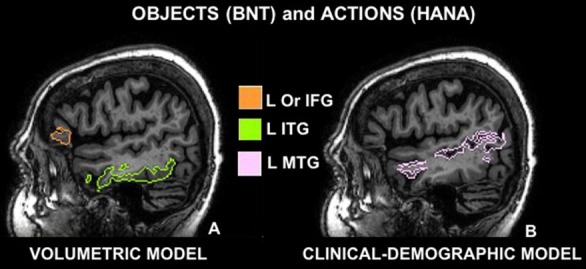
Cerebral areas involved in naming objects (Boston Naming Test, BNT) and actions (Hopkins Assessment on Naming Actions, HANA) before (*volumetric model* **A**) and after controlling for demographic and clinical features (*clinical-demographic model* **B**): **(A)** sagittal view of the left inferior frontal gyrus pars orbitalis (Or IFG) in orange and the left inferior temporal gyrus (ITG) in green; **(B)** sagittal view of the left middle temporal gyrus (L MTG) in pink; according to the multi-atlas labeling of one representative case.

Stepwise regression demonstrated that in the *volumetric* model, with ROIs as the only predictors, left ITG and left IFG *orbitalis* cumulatively accounted for 36% of the total variance in BNT, and no other ROIs increased the R-square. In descending order of variance, left ITG accounted for 28% with a positive correlation—smaller volume in this cerebral area corresponded to worse performance (see Figure 1).

The left IFG *orbitalis* accounted for an additional 8% with a negative correlation—smaller volume corresponded to better performance. Therefore, patients with smaller IFG volumes were performing better in noun naming (Table 2A).

When demographic and clinical features were added in the *clinical-demographic model*, three components cumulatively accounted for 47% of the total variance. In descending order of variance, language severity accounted for 34% with a negative correlation. Since higher severity scores corresponded to larger impairment, this result means that higher language severity scores are correlated with lower performance in BNT (Table 2B). The left MTG added 13% of variance with positive correlation, i.e., smaller volume corresponds to worse performance (see Table 2B).

**Table 2B:** *Clinical-demographic model on BNT*: naming nouns controlled for normalized volume of the 12 left language areas and their homologs plus demographic (gender, age and education) and clinical features (years post-onset, dementia severity, language severity).

Variable	Fraction of std change in BNT, per 1 unit* change in variable (SE)	*t*-stat	*P*-value	*R*^2^	Added *R*^2^
**Stepwise regression of each ROIs, demographic and clinical features**					
Language severity	−0.90 (0.18)	−5.16	<0.001	34%	34%
L MTG	0.55 (0.14)	−3.89	<0.001	47%	+13%
**Stepwise regression of each ROIs, demographic and clinical features, including variant**					
Language severity	−0.99 (0.16)	−6.14	<0.001	34%	34%
Sv variant	−2.34 (0.35)	−6.76	<0.001	52%	+18%
Lv variant	−0.97 (0.36)	−2.66	0.012	55%	+3%
Edu	−0.15 (0.05)	−2.70	0.011	59%	+4%

*Units are in 1 std of variables except for the indicators of variants Sv, Lv. Abbreviations: Language severity, severity score at the language subtest of the FTLD–CDR; BNT, Boston Naming Test, left inferior frontal gyrus pars orbitalis; L MTG, left middle temporal gyrus; Sv, semantic variant PPA; Lv, logopenic variant PPA; Edu, years of education. The Added R-squared from the regression model refers to the additional variance explained by including the given variable.

However, when information about the variant type was added as a factor in the *clinical-demographic model*, the contribution of language severity remained unchanged and, importantly, type of variant and education were significant, accounting for more than half of the variance. Also, no brain areas are left as significant predictors when variant information is added in the model. In descending order of variance, language severity contribution remained the same (34%), semantic variant contributed to 18%, logopenic variant to 3% and education to 4% of added variance explained by the model. The negative sign for the semantic and logopenic variants is to be construed with regard to the reference variable (chosen by the model, in this case the non-fluent variant) and means that these two variants performed worse than the non-fluent variant in object naming.

### Results of Action Naming (HANA)

In the *volumetric model*, where ROIs volumes were the only predictors of performance, only a small percentage of variance in performance was explained, i.e., left ITG explained 14% of the total variance with a positive correlation, meaning that smaller brain volume in this area corresponded to worse performance (see Table 3A, Figure 1). An additional 6% of the variance was explained by the volume of the left IFG *orbitalis*, meaning that patients with smaller left IFG volumes performed better in action naming as was the case in object naming as well.

When clinical and demographic features were added in the analysis, the *clinical-demographic model* captured two components that cumulatively accounted for 49% of the total variance. In descending order, language severity accounted for 42% with a negative correlation (high severity scores corresponded to low performance); the left MTG accounted for an additional 7% with a positive correlation (less volume corresponded to worse performance; Tables 3A,B).

**Table 3B:** *Clinical-demographic model on HANA*: naming verbs controlled for normalized volume of the 12 left language areas and their homologs plus demographic (gender, age and education) and clinical features (years post-onset, dementia severity and language severity).

Variable	Fraction of std change in BNT, per 1 unit* change in variable (SE)	*t*-stat	*P*-value	*R*^2^	Added *R*^2^
**Stepwise regression of each ROIs, demographic and clinical features**					
Language severity	−1.03 (0.17)	6.22	<0.001	42%	42%
L MTG	0.45 (0.13)	−3.53	0.001	49%	+7%
**Stepwise regression of each ROIs, demographic and clinical features, including variant**					
Language severity	−1.07 (0.14)	−7.80	<0.001	42%	42%
Non-fluent variant	1.42 (0.35)	4.07	<0.001	51%	+9%
Logopenic variant	0.67 (0.36)	1.84	0.074	52%	+1%

*Units are in 1 std of variables except for the indicators of variants Nfv, Lv. Abbreviations: Language severity, severity score at the language subtest of the FTLD–CDR; L MTG, left middle temporal gyrus; Nfv, non-fluent variant PPA; Lv, logopenic variant PPA. The Added R-squared from the regression model refers to the additional variance explained by including the given variable.

When information about variants was entered in the regression model, it explained an additional 2% of the variance with regard to the *clinical-demographic* model without variants. The variant information *per se* explained an additional 9% to the 42% of language severity. No brain areas explained any significant percentage of variance in this model. When variant was entered as a separate predictor, the regression showed that the non-fluent variant contributed a significant 9% and the logopenic variant contributed 1% to the variance explained but this additional percent was not significant. The sign for the contribution of both these variants was positive, meaning that non-fluent and logopenic variant participants performed better in verb naming compared to semantic variant participants.

## Discussion

The present study investigated whether demographic and clinical features predict performance on object and action naming more than atrophy in patients with PPA. First, we replicated the significant contribution of the left ITG, as determined in our previous study (Race et al., 2013); this was the only area of the extended language network and its homologs that was correlated with naming performance in both nouns and verbs. Second, we were able to determine the amount of variance in naming performance of objects and actions, attributed to the left ITG. Third, we determined the contribution of other demographic and clinical factors (such as language severity and education) in object and action naming performance in PPA and we discuss them with regard to PPA variant as well. We showed numerically that in complex clinical syndromes, brain volumes cannot explain adequate variance of language performance especially for actions (verbs) and other clinical and demographic factors may explain more variance in naming performance.

In summary, the *volumetric* model explained 36% of the total variance in BNT performance (left ITG 28%, left IFG *orbitalis* 8%), whereas the *clinical-demographic* model explained an additional 11% (language severity 34%, and left MTG 13%; Tables 2A,B). When type of variant was introduced as an additional factor, the *clinical-demographic* model explained 12% more of the variance compared to the previous *clinical-demographic* model: language severity (34%), svPPA (18%), lvPPA (3%) and education (4%) became significant too in place of the volumetric components (Tables 2A,B). In action naming, the *volumetric* model explained 20% of the total variance in HANA performance (with left ITG explaining 14% and left IFG *orbitalis* 6%), whereas the *clinical-demographic* model (without the variants factor) explained an additional 29% (language severity 42%, left MTG 7%). When type of variant was added to the *clinical-demographic* model, it explained an additional 2% of the variance (Tables 2B, 3B). These results highlight the roles of the left ITG and the left MTG in picture naming of objects and actions but also emphasize the importance of clinical factors such as language severity in naming performance. Below we discuss the implications of the above findings.

### The Contributions of the Left ITG, Left MTG and Left IFG in Naming Objects and Actions

As in our previous study (Race et al., 2013), the left ITG was shown to be the most significant area in predicting naming performance in both objects and actions in PPA since this was the only area in which degree of atrophy was positively and significantly correlated with naming performance. This is an area involved in a variety of lexical tasks, including naming (Price and Devlin, 2003). Many studies have demonstrated the importance of the left ITG for object and action naming as well as other tasks requiring lexical retrieval (Moore and Price, 1999; Hillis et al., 2006a; Race et al., 2013; Sebastian et al., 2014). In the brain parcellations used in this study, the left ITG corresponds to BA 20 and BA 37. The critical role of this area in object naming has also been demonstrated in stroke recovery after reperfusion of the left ITG that resulted in improvement of naming scores in acute stroke (Hillis et al., 2006b).

Interestingly, in the present study, object and action naming performance was no longer predicted by the inferior temporal cortex when controlling for the severity of language deficits. Therefore, it seems that the variance explained by the left ITG in the *volumetric* model was probably absorbed by language severity in the *clinical-demographic* model, verifying the high correlation of language severity to atrophy in the left ITG.

With a small but still significant percentage of variance explained compared to the other predictors, the volume of the left MTG became significant in the naming performance of objects and actions after controlling for clinical features, demonstrating that its involvement was independent from language severity. Left MTG relevance in the retrieval of objects has already been confirmed by lesion studies with aphasic patients (Damasio and Tranel, 1993; Daniele et al., 1994; Tranel et al., 2001; Hillis et al., 2002a). The analyses in the present study showed that the contributions of the left ITG and left MTG in naming may be independent from each other. Therefore, temporal areas might represent the key area for naming across PPA variants. The present study demonstrated that the left MTG (compared to the left ITG) is involved in naming of objects and actions independently of the degree of language impairment (language severity measured by the FTD–CDR scale). The present findings indicate that naming deficits in all PPA variants may also be due to atrophy in the inferior (Race et al., 2013), as well as in the middle temporal regions independent from severity, even though these are not the primary areas of atrophy in any of the variants.

The negative correlation of the frontal area with BNT scores described an inverse correlation between the left IFG *orbitalis* volume and performance on naming nouns, meaning that smaller frontal volume corresponded to better performance. A possible explanation of this result is that it may be driven by non-fluent participants (nfvPPA) who—despite their atrophy in frontal areas and motor-speech deficits (Grossman et al., 1996; Gorno-Tempini et al., 2004)—seem to have fewer word-retrieval deficits considered here (see Table 1). We tested this hypothesis by removing the nfvPPA participants from the data and re-estimated the model with the variables shown in Table 2B. In this analysis, the added R-square of the left IFG *orbitalis* was less than 1% and non-significant. As indicated in other studies as well (Thompson et al., 2012), participants with nfvPPA show better performance in naming compared to those with lvPPA and svPPA (Henry et al., 2015). Furthermore, when language severity is entered as a predictor in the *clinical-demographic* model(s), the contribution of the left IFG disappears for both object and action naming.

### The Role of Language Severity and Variant in Naming Performance

The *clinical-demographic* model added 11% (Table 2B) of the variance explained in object naming compared to the *volumetric* model (Table 2A), with severity of language explaining the highest percentage of variance (34%) in both *clinical-demographic* models. Similarly, in naming actions, the *clinical-demographic* model (Table 3B) added 29% of the variance explained compared to the *volumetric* model (Table 3A), i.e., to the 14% of the left ITG and the 6% of the left IFG orbitalis that were the only areas of significant contribution to variance. Language severity was still the only clinical factor that contributed highly to the variance in object naming (34%), as well as in action naming (42%), and it seems it absorbed the variance attributed to the left ITG and left IFG orbitalis in the *volumetric* model for both objects (28% and 8%) and actions (14% and 6%). These findings highlight the critical role of language severity ratings in explaining object and action naming. This finding raises some interesting reflections about the correlation between the degree of atrophy of the ITG and IFG areas and the level of disease severity evaluated in the clinical assessment. One can speculate that these areas might not benefit from cognitive reserve; instead they might present a positive correlation between the thickness of their cerebral volume and the manifestation of the cognitive symptoms.

The significant increase of variance explained by clinical factors in the *clinical-demographic* vs. *volumetric* models for both object and action naming that was even steeper in actions (from 20% to 49%) highlight the multifactorial nature of naming performance. It also shows that verb naming is more complex than noun naming since verbs have more complex semantics, morphology and syntax than nouns (especially in English); therefore, verbs are more susceptible to deterioration with disease progression (Thompson and Mack, 2014) and this may warrant verbs being a significant rehabilitation target.

We would like to make a specific note about the contribution of variant type in noun and verb naming in PPA. When we included the variant information in the regression of the *clinical-demographic* model, we found that it was the second most significant predictor on both noun and verb naming performance after language severity and, most importantly, it contributed independently from language severity and absorbed all the variance explained by all brain areas. Different variants contributed differently in the naming of nouns and verbs: the low performance of the semantic variant contributed significantly to the variance explained for noun naming, and the high performance of the non-fluent variant contributed significantly to the variance explained for verb naming. These results confirm previous observations about the classification and subtyping of PPA variants (Gorno-Tempini et al., 2011) and show how clinical information related to a diagnosis between variants can be as predictive for naming performance as complex volumetric data. The importance of considering the type of variant as a factor in the *clinical-demographic* model, was shown with the additional variance explained, compared to the previous *clinical-demographic* model without the variant information. This additional analysis confirmed that the performance of svPPA and lvPPA patients in object and verb naming was significantly lower than performance of nfvPPA patients, confirming previous results in the literature (Gorno-Tempini et al., 2004).

### Demographic Considerations in Naming Performance: Gender and Education

In contrast to studies showing that in patients with Alzheimer’s disease (AD) and healthy elderly controls, where men performed significantly better than women in naming objects (Randolph et al., 1999; Hall et al., 2012), our study did not provide support for any gender or age effects shown in previous studies (Randolph et al., 1999) in either object or action naming. The absence of a significant age or gender effect in our study aligns with the study by Rogalski et al. (2007) on a different large cohort of PPA patients. A possible explanation for these results is that PPA is a heterogeneous syndrome, making it difficult for gender or age effects to be detected. Alternatively, there may exist an interaction between gender effects and language severity, i.e., gender effects may be apparent only at earlier stages as Hall et al. (2012) showed, or they may be related to a specific pathology such as AD and thus were not apparent in our sample which probably included people with several pathologies. Both Rogalski et al. (2007) and our study included participants with variable levels of language severity and found no gender effects. Our current findings suggest that these features (age, onset, and gender) are not significantly associated with naming performance in PPA patients.

Another interesting finding was the small but significant contribution of education (4%), showing that PPA patients’ performance on naming objects was not associated with poor education, but instead that more years of schooling correlated with lower performance. This finding probably depicts the effect of poor naming in PPA patients who nevertheless had high education (most patients in our sample were generally well-educated, i.e., above 16 years), and there was no range in education as seen in AD samples. In stroke patients, education provided resilience in particular for written naming, compared to oral naming of objects (González-Fernández et al., 2011). The small but significant effect of education for object naming that was independent from language severity in our cohort may reflect the fact that this well-educated sample may indeed manifest the neuroprotective role of education: those who were recruited due to poor naming had higher education because high education had not allowed the disease to manifest or progress until that point.

### Limitations of the Present Study

One limitation of this study is that it focused mainly on lexical processes involved in picture naming. A seemingly simple task such as naming objects or actions involves at least three cognitive components of the language production system: retrieval of the meaning from the conceptual system, retrieval of the phonological representation of the word in the lexicon, and then coordination of the orofacial, palatal, jaw, laryngeal and respiratory muscles to produce the word, according to models by Caramazza (1997) and Levelt (1999). Therefore, the same deficit, such as failure to name a picture, may result from a deficit in the semantic system in svPPA, a deficit in searching the lexicon in lvPPA, or a deficit in coordinating muscles in nfvPPA. By not penalizing motor-speech errors, we focused predominantly on access to meaning and access to the lexicon and phonological representations but not on motor-speech schemata. Another limitation of the present study is that we did not use a voxel-based approach to determine atrophy but instead we used an atlas where areas are defined cytoarchitectonically and therefore the contribution of small areas or significant divisions of larger areas may not have reached significance. Finally, we used only gray matter volume to see correlations with naming performance and not any white matter measures or functional measures, so the contribution of such measurements to naming was not captured.

## Author Contributions

MR, CO, JD, CF and KT contributed to the conception and design. BF and KW acquired the data. MR, AF, CO, CF and KT analyzed and interpreted the data. MR, AF, BF, KW, CF and KT drafted and/or revised the article critically for intellectual content.

## Conflict of Interest Statement

The authors declare that the research was conducted in the absence of any commercial or financial relationships that could be construed as a potential conflict of interest.

## Footnotes

^1^www.MRICloud.org

^2^https://masi.vuse.vanderbilt.edu/workshop2013/images/1/1b/SATA_2013_Proceedings.pdf
